# Elucidation of an Aggregate Excited State in the Electrochemiluminescence and Chemiluminescence of a Thermally Activated Delayed Fluorescence (TADF) Emitter

**DOI:** 10.1021/acs.langmuir.2c03391

**Published:** 2023-02-10

**Authors:** Kenneth Chu, Jonathan R. Adsetts, Zackry Whitworth, Shiv Kumar, Eli Zysman-Colman, Zhifeng Ding

**Affiliations:** † Department of Chemistry, Western University, London, ON N6A 5B7, Canada; ‡ Organic Semiconductor Centre, EaStCHEM School of Chemistry, University of St. Andrews, St. Andrews, Fife KY16 9ST, U.K.

## Abstract

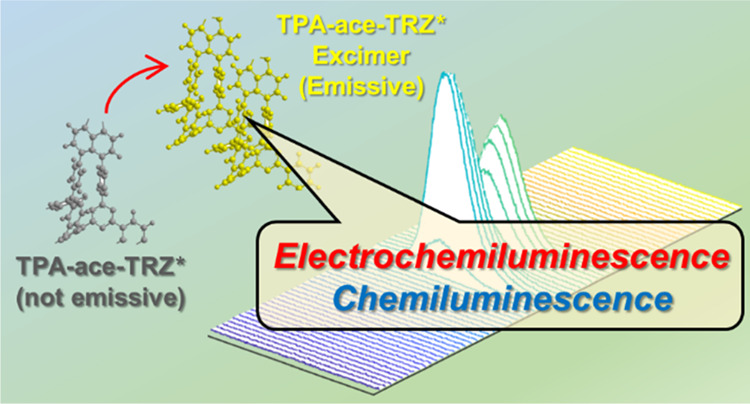

The electrochemistry, electrochemiluminescence (ECL), and chemiluminescence (CL) properties of a thermally activated delayed fluorescence (TADF) emitter 4,4′-(1,2-dihydroacenaphthylene-5,6-diyl)bis(*N*,*N*-diphenylaniline) (TPA-ace-TRZ) and three of its analogues were investigated. TPA-ace-TRZ exhibits both (a) delayed onset of ECL and (b) long-persistent luminescence, which we have attributed to the formation of an aggregate excited state in excimer or exciplex form. The evidence of this aggregate excited state was consistent across ECL annihilation and coreactant pathways as well as in CL. The absolute ECL efficiency of TPA-ace-TRZ using benzoyl peroxide (BPO) as a coreactant was found to be 0.028%, which was 9-fold stronger than the [Ru(bpy)_3_]^2+^/BPO reference coereactant system. Furthermore, the absolute CL quantum efficiency of TPA-ace-TRZ was determined to be 0.92%. The performance and flexibility of the TADF emitter TPA-ace-TRZ under these various emissive pathways are highly desirable toward applications in sensing, imaging, and light-emitting devices.

## Introduction

Electrochemiluminescence (ECL) involves the electro-generation of radical species that subsequently undergo electron transfer reactions to form excited states, which release photons upon relaxation.^1,2^ ECL has many analytical applications including biological immunoassays,^3−5^ analyte detection,^6,7^ single-molecule detection,^5,8−10^ and various imaging application,^11,12^ along with various luminophores.^1,2,13−19^ Since ECL does not require an incident light source, detection can be achieved with excellent signal-to-noise ratio and sensitivity.^17^ There are two general pathways by which ECL can occur. The first is the annihilation pathway, where radical species generated by oxidation and reduction at an electrode interact to produce excited states. The second is the coreactant route, which introduces a secondary compound known as a coreactant, such as benzoyl peroxide (BPO). Upon reduction, BPO can form a benzoate radical through electrochemical and chemical reactions that has significant oxidizing power and is capable of oxidizing the luminophore radical anion to produce excited states. Due to the high redox power of coreactants, ECL in coreactant pathways often has greatly enhanced emission.^17^

Chemiluminescence (CL), on the other hand, is another type of luminescence where the excitation energy instead comes from chemical reactions.^20−23^ In many ways, it is a more generalized version of ECL, and so CL-based detection techniques enjoy many of the same analytical benefits. One of the most studied CL reactions is the oxidation of an aryl oxalate ester with hydrogen peroxide; this reaction produces high-energy intermediates that are capable of chemically exciting luminophores;^24−26^ radiative relaxation back to ground state releases energy in the form of light. Excited-state species may also lose their energy by other mechanisms such as vibrational relaxation and collisions with other molecules; such nonradiative processes lead to decreased CL emission efficiency.^27,28^

Numerous classes of ECL and CL luminophores have since been studied, including organic molecules,^29,30^ phosphorescent metallic complexes,^31,32^ nanomaterials,^15,18,19,33,34^ and their emulsion droplets^35^ like PL of luminophore aggregate at liquid/liquid interfaces.^36^ Thermally activated delayed fluorescent (TADF) emitters are a new class of luminophores in ECL able to utilize thermally activated upconversion of triplet to singlet states, thus enabling theoretical internal quantum efficiencies of up to 100%.^37^ TADF relies on a small singlet–triplet energy gap, Δ*E*_ST_. More recently, several reports have documented organic long-persistent luminescent compounds whose luminescence decays in the order of seconds.^38,39^ The long-lived luminescence in this class of emitters originates from charge separation, followed by a slow charge recombination route, often in a framework of electron-donating and electron-accepting molecules to facilitate the formation of charge-separated states.^40^ This phenomenon has been reported for photoluminescent materials (OLPL),^41^ but also recently by us for electrochemiluminescence (OLECL).^16,42^ In particular, there is evidence that the compounds that exhibit organic long-persistent luminescence often possess aggregate excited states.^43^

**Figure 1:**
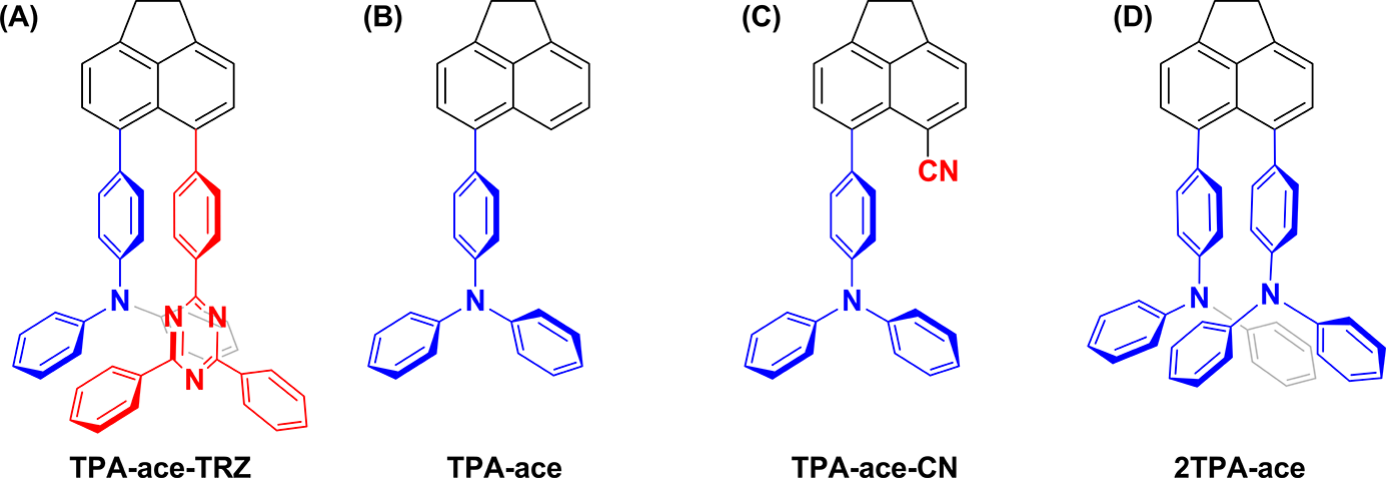
Structures of (A) TPA-ace-TRZ (B) TPA-ace, (C) TPA-ace-CN, and (D) 2TPA-ace.

Recently, the synthesis and photophysical properties of a series of through-space charge transfer thermally activated delayed fluorescence compounds 4,4′-(1,2-dihydroacenaphthylene-5,6-diyl)bis(*N*,*N*-diphenylaniline) (TPA-ace-TRZ) (Figure 1A), 4-(1,2-dihydroacenaphthylen-5-yl)-*N*,*N*-diphenylaniline (TPA-ace) (Figure 1B), 6-(4-(diphenylamino)phenyl)-1,2-dihydroacenaphthylene-5-carbonitrile (TPA-ace-CN) (Figure 1C), and 4,4′-(1,2-dihydroacenaphthylene-5,6-diyl)bis(*N*,*N*-diphenylaniline) (2TPA-ace) (Figure 1D) were first investigated by us.^44^ Due to the intriguing electronic properties of TPA-ace-TRZ, we further explore in this study the electrochemistry, electrochemiluminescence, and chemiluminescence of TPA-ace-TRZ, Figure 1A. Using ECL–voltage curves and time-resolved ECL spectroscopy, we provide unique insights into the formation and emission characteristics into aggregate excited states. Also, the ECL and CL absolute quantum yields were determined for TPA-ace-TRZ, providing valuable electrochemical and spectroscopic insights.

## Experimental Section

### Materials and Reagents

Tetrabutylammonium hexafluorophosphate (TBAPF_6_, for electrochemical analysis, ≥99.0%), benzoyl peroxide (BPO, reagent grade, >98%), and ferrocene (>98%) were purchased from Sigma-Aldrich Canada and used as received. SureSeal dichloromethane (DCM, ≥99.8%) was purchased from Sigma-Aldrich Canada and stored in a N_2_-filled glovebox. The synthesis of the above compounds is reported elsewhere.^44^

### Electrochemistry and Electrochemiluminescence

A three-electrode system was used for all electrochemical measurements, where the working electrode was a 2 mm platinum disk inlaid in a glass tube, and the counter and quasi-reference electrodes were platinum wires. All potentials were reported relative to the Fc/Fc^+^ redox couple where the formal potential was taken to be 0.342 V vs SCE.^45^ Electrochemiluminescence experiments were conducted inside a cylindrical glass tube with a flat quartz window at the bottom to allow for the detection of ECL light. The airtight ECL cell was assembled inside a nitrogen atmosphere glovebox (Model Nexus I, Vacuum Atmospheres Company, Hawthorne, CA) to minimize the effect of oxygen and moisture.

The potentiostat used for cyclic voltammetry and differential pulse voltammetry experiments was a CH Instruments Model 610a electrochemical workstation (CH Instruments, Inc., Austin, TX). ECL emission was measured using a photomultiplier tube (Model R928, Hamamatsu, Japan) biased at −750 V, where the output signal as photocurrent was converted in a voltage for data acquisition using a picoammeter (Keithley 6487, Cleveland, OH). The electrochemical current and the ECL signal were recorded using a data acquisition board (DAQ Model 6036E, National Instruments, Austin, TX) and acquired using a custom LabVIEW program. For potential stepping experiments, a PAR263 potentiostat was utilized (Princeton Applied Research, Berwyn, PA), which also recorded the ECL signal by means of an external auxiliary input. ECL spectra were recorded using a spectrograph (Model SP2300i, Princeton Instruments, Trenton, NJ) with an attached CCD camera (Andor DU401-BR-DD-352, Oxford Instruments, UK) cooled to −65 °C. Wavelength calibration was accomplished using a mercury source (HG-1, Ocean Optics, Dunedin, FL) using a center wavelength of 546 nm. Accumulation ECL spectra were acquired by collecting all emission generated over the entire cyclic voltammogram program. Spooling ECL spectra were acquired each at a time interval of 1 s during a cyclic voltammogram; the obtained spectra were combined in a three-dimensional plot using a custom MATLAB program. For all measurements, the spectrum recording was synchronized by means of a 5 V TTL pulse output from the potentiostat at the beginning of the potential scanning.
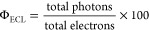
1

### Determination of the Absolute ECL Quantum Efficiency

The spectrograph/CCD camera setup described above was standardized against a calibrated photodiode (S120VC, Thorlabs Optics, Newton, NJ) and power meter (PM100D, Thorlabs). Following this calibration procedure, the reading from the CCD camera, in *counts*, may be converted to an absolute quantity, in *photons*. In-depth experimental procedures and calibration formulas are described in detail elsewhere.^46,47^ Determination of the total number of electrons injected during an experiment was performed by integrating the electrochemical current (as measured by the electrochemical workstation) to obtain the total charge; transformation to number of electrons proceeds using the *elementary charge constant*. The absolute ECL quantum efficiency (Φ_ECL_) is then determined using eq 1.

### Chemiluminescence

**Figure 2:**
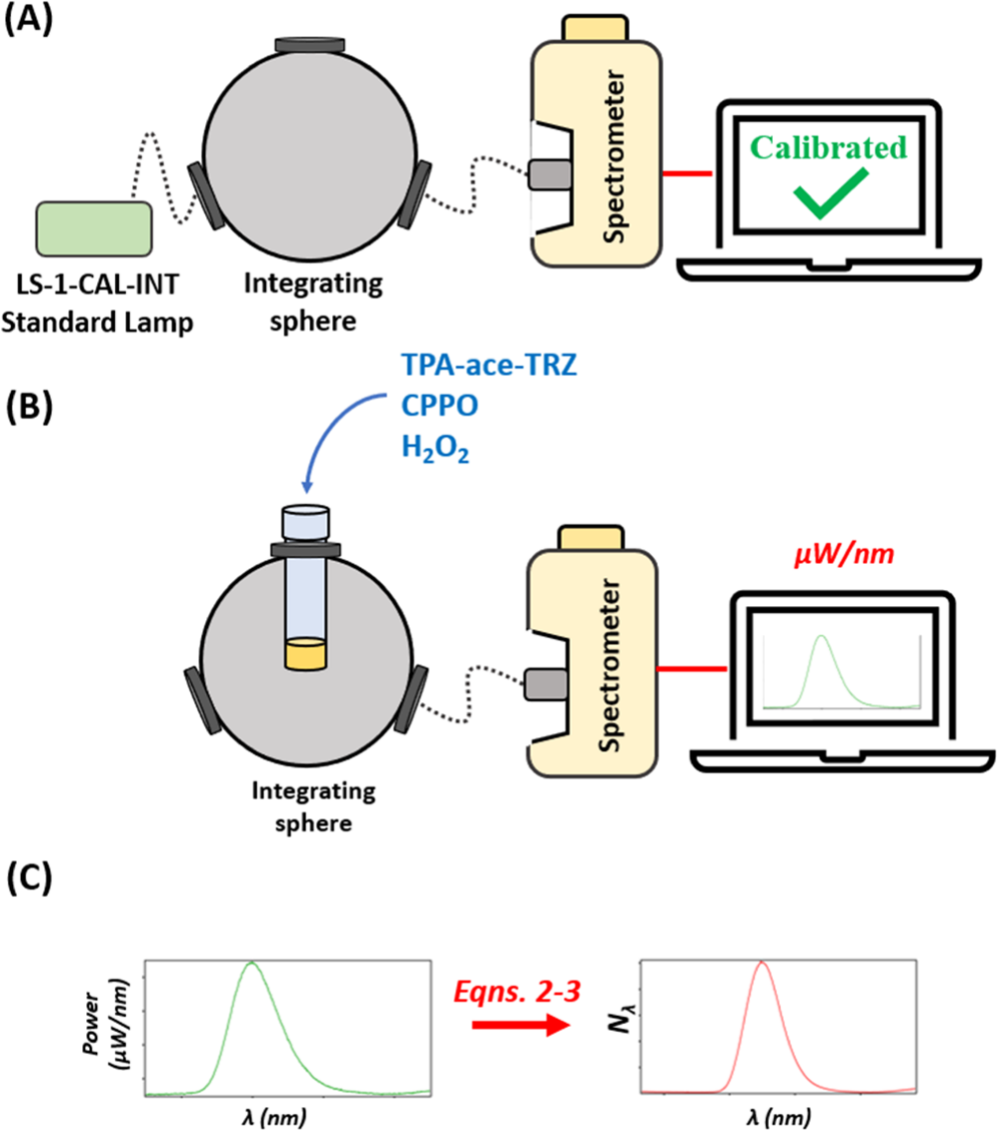
(A) Illustrative calibration procedure of the spectrometer and integrating sphere detection system. The LS-1-CAL-INT standard lamp was radiometrically calibrated by Ocean Insight, Inc. to provide a known quantity of light. The lamp is automatically tracked by the OceanView software to provide the absolute irradiance power spectrum (power in μW/nm vs wavelength in nm). (B) Measuring the absolute chemiluminescence (CL) quantum efficiency. The CL reagents are added to the reaction vial, and the resulting emission is collected in absolute photon numbers by the calibrated spectrometer system. (C) Transformation from absolute power (μW/nm) to absolute number of photons (1/nm) at each individual wavelength, using eqs 2–3. The number of total photons can then be obtained by summing up the photons at each wavelength in the visible range between 400 and 950 nm. Dotted lines in the figure correspond to connections via optic fibers.

Chemiluminescence experiments were performed by adding the following reagents into the reaction vial: 10 mL of ethyl acetate (reagent grade, >99.5%, Sigma-Aldrich Canada) as the solvent, 50 mg of bis(2,4,5-trichloro-6-(pentyloxycarbonyl)phenyl)oxalate (CPPO, >98%), 100 mg of sodium acetate, and 3 mL of 30% hydrogen peroxide (H_2_O_2_). This recipe was adapted from a paper^48^ with some modifications in consideration of the reagent solubility. The luminophore (TPA-ace-TRZ) was added at a concentration of 0.3 mg/mL. Spooling CL spectra were collected using a 6-inch integrating sphere (Labsphere Inc., North Sutton, NH). An optical fiber connected the integrating sphere to an optical spectrograph and sensor (USB2000+, Ocean Insight, Orlando, FL) which was controlled by OceanView software (Ocean Insight). Calibration of the Ocean Insight spectrometer was performed using a radiometric standard lamp (Model LS-1-CAL-INT, Ocean Insight), as in Figure 2A. Determination of the chemiluminescence absolute efficiency (Φ_CL_) was performed by converting an absolute irradiance power spectrum (*W*_λ_ in μW/nm vs wavelength in nm) from the Ocean Insight spectrometer to an absolute photon spectrum (*N*_λ_ in photons/nm vs wavelength in nm) using eqs 2 and 3, Figure 2B.
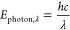
2
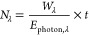
3where *E*_photon,λ_ is the energy of a photon at a specific wavelength, *h* is Planck’s constant, *c* is the speed of light, λ is the wavelength, and *t* is the accumulation time for acquiring the spectrum. Following eq 4 by summing up all of the photons at each wavelength in the range of 400–950 nm for all spooling CL spectra, the absolute CL efficiency (Φ_CL_) can be determined
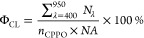
4where *n*_CPPO_ is the number of CPPO molecules (the limiting reagent) in the reaction and *NA* is Avogadro’s constant (equal to 6.02 × 10^23^).

## Results and Discussion

### Electrochemiluminescence via the Annihilation Pathway

The electrochemical and spectroscopic properties of the four complexes (TPA-ace-TRZ, TPA-ace, CN-TPA-ace, and 2TPA-ace) were studied. However, due to the very small singlet–triplet energy gap (Δ*E*_ST_) of TPA-ace-TRZ (0.06 eV), we decided to investigate this compound in more detail, as the small Δ*E*_ST_ could lead to enhanced electrochemiluminescence efficiencies through effective harvesting of triplet excitons.^49−51^

**Figure 3:**
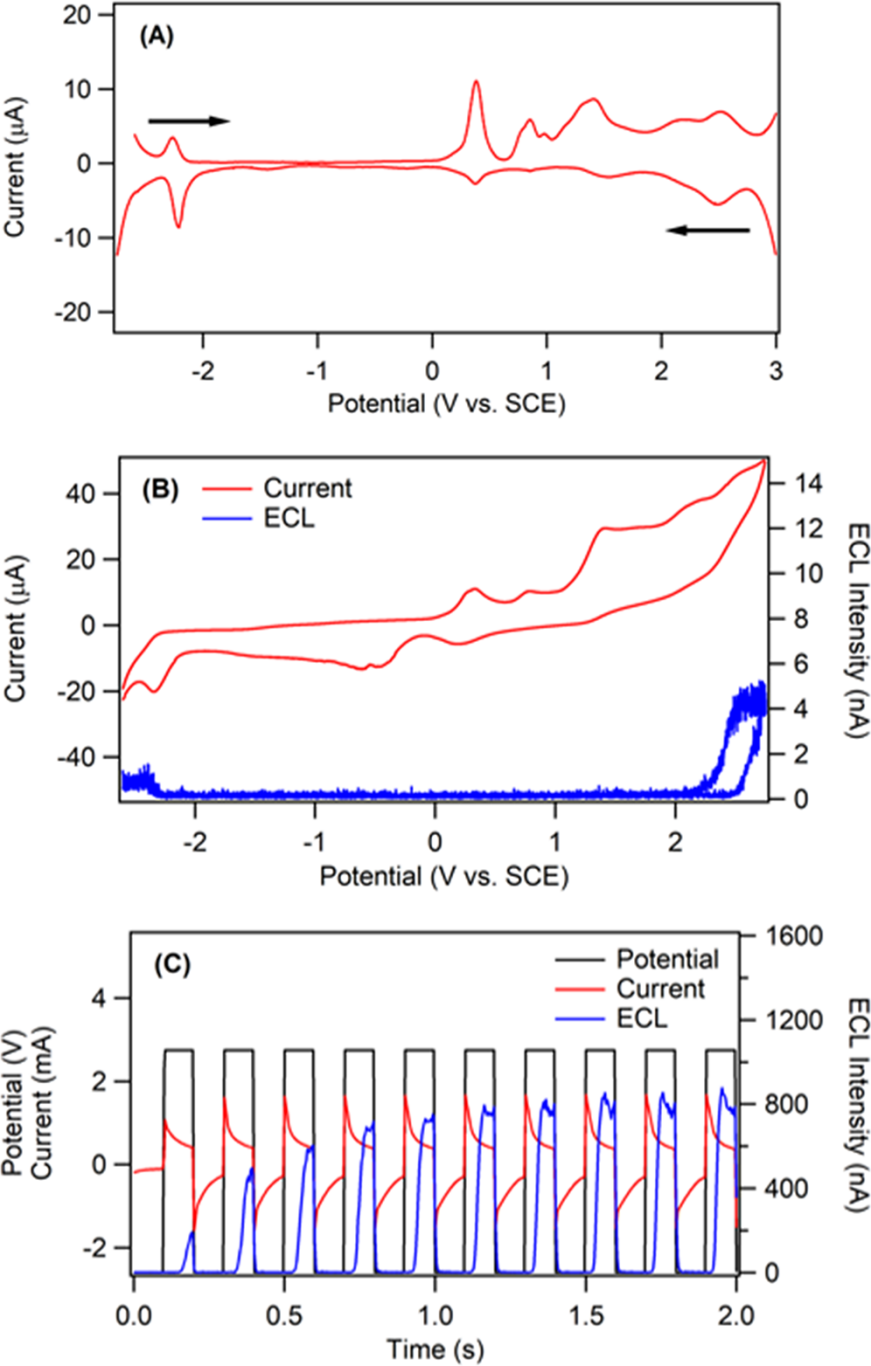
(A) Differential pulse voltammogram of 1.42 mM TPA-ace-TRZ in dichloromethane. Initial scan direction is indicated with arrows. (B) CV (red) and the corresponding ECL–voltage curve (blue) of 1.42 mM TPA-ace-TRZ in dichloromethane with 0.1 M TBAPF_6_ as supporting electrolyte. Scan rate was 0.1 V/s. (C) Potential–time (black), current–time (red), and ECL–time (blue) profiles of TPA-ace-TRZ during potential stepping experiments at a pulsing frequency of 10 Hz.

The electrochemistry and the ECL behavior of TPA-ace-TRZ in the annihilation pathway were first investigated. Figure 3A displays the differential pulse voltammograms (DPVs) of TPA-ace-TRZ in dichloromethane, while Figure 3B shows the cyclic voltammogram (red) and corresponding ECL–voltage curve (blue). TPA-ace-TRZ undergoes multiple irreversible oxidation reactions and one quasi-reversible reduction reaction at −2.20 V as seen in the DPVs. One notable oxidation occurs at +0.50 V. However, ECL was only observed when the potential reached the oxidation peak at +2.30 V and the reduction peak at −2.20 V vs. SCE. This suggests that the generation of the radical anion, and at least a radical multication of TPA-ace-TRZ is required for ECL. The intensity of ECL detected in the annihilation pathway was limited, with a maximum of 5 nA observed in the anodic scan. This is likely due to the low stability of the electrogenerated radical species; this issue is especially apparent in linear voltage sweep experiments, as there is a relatively long time gap between the generations of radical ion partners. It can also be seen that the ECL is more intense in the anodic region, which suggests that TPA-ace-TRZ^•–^ possesses greater stability compared to TPA-ace-TRZ^•+^, which agrees well with the reversibility of redox reactions.

The problem of large time delay between generations of radical anions and cations can be partially circumvented with potential stepping experiments, where the potential can be rapidly switched between anodic and cathodic voltages. Figure 3C shows the ECL–time curve for TPA-ace-TRZ in the annihilation pathway (ECL plotted in blue), where the potential was alternated between +2.75 and −2.65 V at a rate of 10 Hz. The ECL intensity was greatly enhanced using this process, and a maximum of 800 nA could be observed during the anodic pulses. This intense ECL enabled us to acquire an accumulation spectrum of TPA-ace-TRZ in the annihilation pathway (Figure S1), where an emission peak centered at 630 nm was observed. The ECL efficiency of this system relative to [Ru(bpy)_3_]^2+^ was determined to be 3.4%. The electrochemistry and annihilation ECL behavior in the same system was also studied for TPA-ace, TPA-ace-CN, and 2TPA-ace. Figures S2–S13 in the Supporting information provide the CV/ECL–voltage curves, DPVs, ECL pulsing profiles, and ECL annihilation accumulation spectra for these three compounds. The emission wavelengths were determined to be 595, 509, and 625 nm, respectively. The summary of the various emission pathways studied for these compounds is provided in Table 1. The ECL emission is significantly red-shifted for 2TPA-ace and TPA-ace-TRZ compared to their PL emissions; this observation may be due to the formation of aggregate excited states, which form as a result of a reaction between two chromophores. These *excimers*—dimeric excited states—may be responsible for the red-shifted emission due to their greater degree of conjugation.^52−54^

**Table 1:** Summary of Photoluminescence (PL), Electrochemiluminescence (ECL), and Chemiluminescence (CL) Emission in Anhydrous Dichloromethane^a^

	TPA-ace-TRZ	TPA-ace-CN	2TPA-ace	TPA-ace
Abs	340 nm	340 nm	340 nm	330 nm
PL	565 nm	495 nm	405 nm	410 nm
ECL annihilation	630 nm (3%)	509 nm (7%)	625 nm (15%)	595 nm (3%)
ECL (10 mM BPO)	610 nm (2200%)	500 nm (2322%)	625 nm (1351%)	605 nm (86%)

a Numbers in parentheses in red color represent ECL efficiencies calculated relative to the Ru(bpy)_3_^2+^/BPO system.

We also observed a noticeable delay in the onset of the ECL signal during potential stepping experiments; this can be seen as the ECL begins to increase approximately 25 ms after each anodic step. The delay in the onset of ECL is therefore the time required to form the emissive excimer species. Table S1 provides a summary of the ECL onset, ECL maximum, and the ECL decay profiles of TPA-ace-TRZ, TPA-ace, 2TPA-ace, and TPA-ace-CN in the ion-annihilation pathway. In all four compounds studied, there was a delay in both the onset of ECL and its decay back to baseline, i.e., ECL started to be detected after the beginning of the potential pulse, and ECL continued to persist after the potential pulse had ended. The former observation is, as stated, due to the required formation of emissive excimer species; the latter might be the phenomenon of *organic long-persistent ECL* (OLECL), which is characterized by long-lived emission stemming from a charge separation process followed by a slow charge recombination route.^41,55,56^ The formation of higher-order excited state species may be characterized by long-persistent luminescence.^43^ Importantly, organic long-persistent emission is mechanically distinct from phosphorescence processes: organic long-persistent emission involves a slow charge recombination step, whereas phosphorescence requires a slow radiative transition between the triplet excited state and the ground state.^57−59^ On average, we observed that this ECL onset delay and persistent ECL was longest for TPA-ace-TRZ, suggesting a slower excimer formation process for this compound compared to the others.

### Electrochemiluminescence with Benzoyl Peroxide as Coreactant

**Figure 4:**
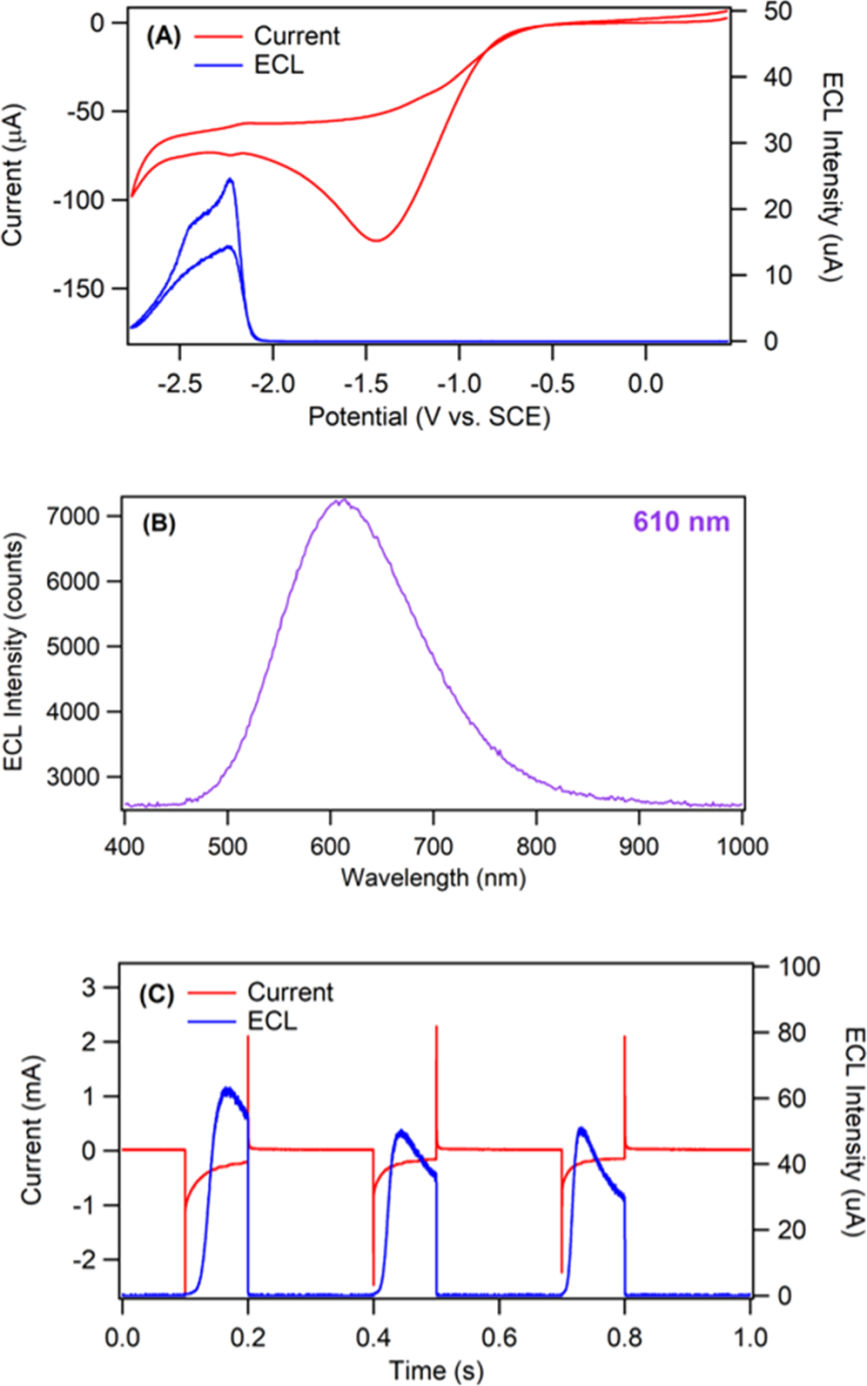
(A) Cyclic voltammogram (red) and ECL–voltage curve (blue) of 1.42 mM TPA-ace-TRZ in dichloromethane and 0.1 M TBAPF_6_ with 10 mM benzoyl peroxide as coreactant. Scan rate = 0.1 V/s. (B) ECL accumulation spectrum of TPA-ace-TRZ with 10 mM benzoyl peroxide (BPO). (C) Current–time (red) and ECL–time (blue) profile of TPA-ace-TRZ during potential pulsing (pulsing frequency was 10 Hz).

Next, we studied the ECL behavior in the presence of a coreactant. Since Figure 3B indicates that the radical anion species is more stable, we selected benzoyl peroxide (BPO) as the oxidative-reduction coreactant. At a concentration of 10 mM BPO, a large enhancement in the ECL intensity was observed, which is due to the close potentials at which luminophore and coreactant can be reduced. In the above scenario, the two radical species react immediately upon generation so that the excited state concentration is elevated. Figure 4A demonstrates the CV and corresponding ECL–voltage curve, where BPO is reduced at around −1.40 V vs. SCE to form the BPO radical anion (BPO^•–^). The benzoate radical (PhCO_2_^•^) that is subsequently formed via a chemical reaction is a strong oxidizing agent which is capable of removing an electron from the TPA-ace-TRZ^•–^ radical anion to generate the excited state TPA-ace-TRZ* that relaxes to emit an ECL photon. The onset of the ECL peak is observed at −2.10 V vs. SCE, which corresponds closely with the reduction of TPA-ace-TRZ (seen from the DPVs in Figure 3A). This confirms that the TPA-ace-TRZ radical anion is key to the production of ECL. The ECL signal reached a maximum intensity of 24 μA at −2.20 V (approximately a 4800-fold enhancement compared with that in the annihilation pathway), with the intensity decreasing at higher applied potentials due to the depletion of excited state concentration. Figure 4B illustrates the ECL emission spectrum of the TPA-ace-TRZ/BPO system, where a peak centered at 610 nm can be observed. This ECL emission wavelength under the coreactant pathway closely matches that of the annihilation pathway, indicating that a similar excited state could be present in both ECL pathways.

Figure 4C shows the ECL–time profile for TPA-ace-TRZ during potential stepping experiments in the BPO coreactant pathway. Similar to what was detected in the annihilation pathway, there was a delay in the onset of ECL emission compared to the beginning of the cathodic potential step. This observation strongly indicates the formation of a higher-order excited state. The production of an *exciplex* species (a heterogeneous excited state^60,61^) between TPA-ace-TRZ^–•^ and PhCO_2_^•^ could be responsible for light emission in the coreactant pathway. This presence of exciplexes could also explain the small difference in emission wavelengths between the annihilation and coreactant pathways.

The ECL behavior in the BPO coreactant system was also studied for TPA-ace, TPA-ace-CN, and 2TPA-ace; Figures S14–S22 demonstrate the CV/ECL–voltage curves, accumulation, and spooling ECL spectra for these experiments. The emission wavelengths for TPA-ace, TPA-ace-CN, and 2TPA-ace were 500, 625, and 605 nm, respectively, which correspond very well to the annihilation ECL emission peak maxima. In general, the ECL emission was strongest for TPA-ace-TRZ and TPA-ace-CN, with an ECL maximum of 24 and 50 μA observed, respectively. In all four studied complexes, we observed a redshift in their ECL emissions compared to their PL emissions; this can again be attributed to the formation of exciplexes.

**Figure 5:**
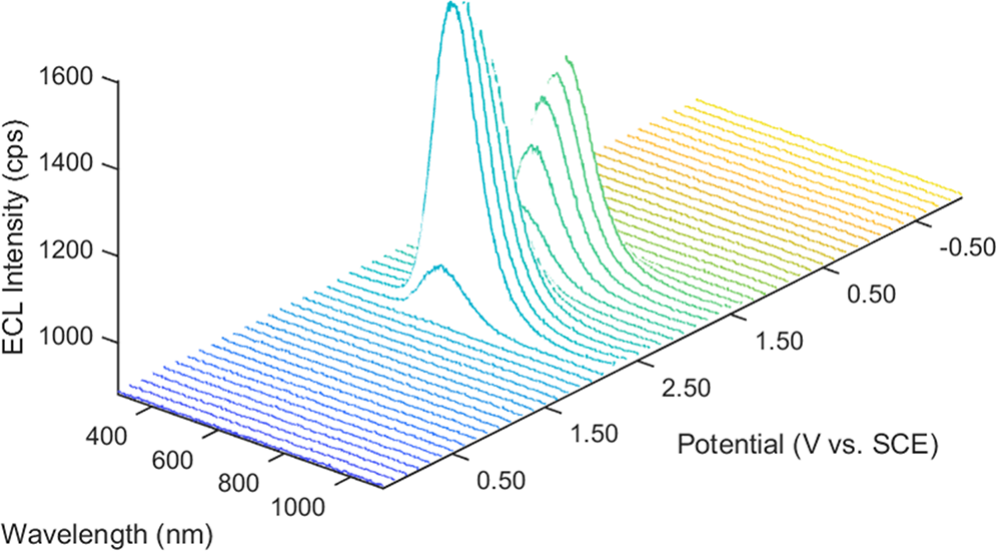
Spooling ECL spectra of TPA-ace-TRZ in the presence of 10 mM BPO as coreactant during a potentiodynamic experiment. The scan rate was 0.1 V/s, and the exposure time of each spectrum was 1 s.

**Figure 6:**
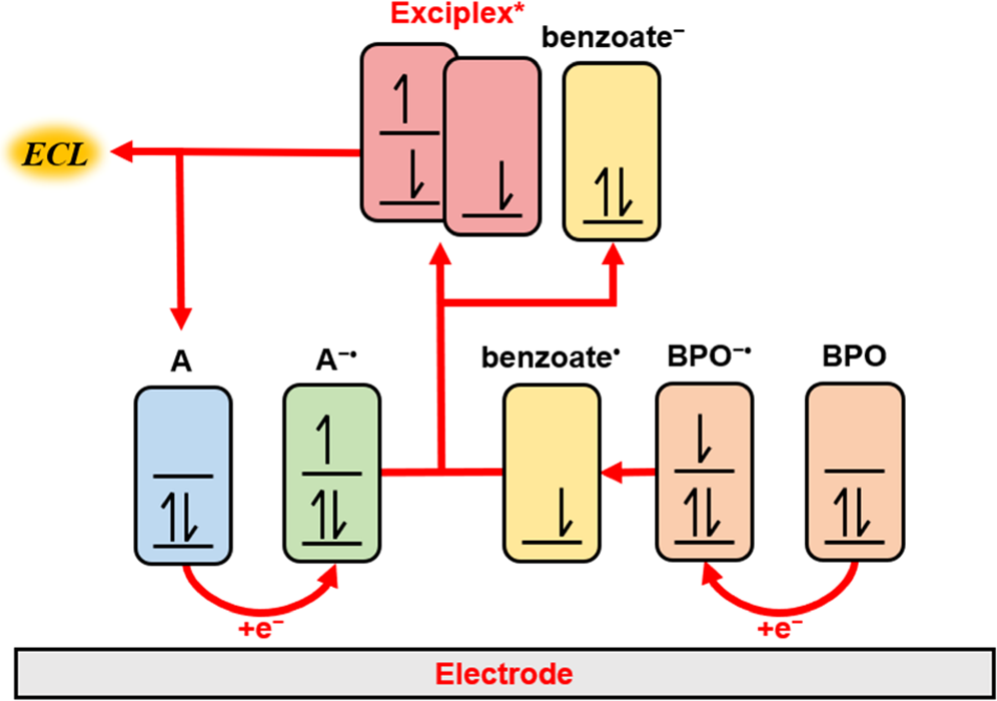
ECL reaction scheme for luminophore in the presence of BPO as coreactant. A = TPA-ace-TRZ, BPO = benzoyl peroxide.

We then employed spooling ECL spectroscopy to further study the emission during a potentiodynamic scan. In this technique, ECL spectra are continuously collected during a voltage scan to enable the correlation of light emission with specific applied potentials.^62^Figure 5 shows the spooling spectra for TPA-ace-TRZ with 10 mM BPO. Like Figure 4A, ECL emission is detected when the potential reaches −2.10 V, with the maximum emission intensity achieved at a potential of −2.20 V. We can see a single peak throughout the scan with the wavelength remaining consistent, shown in the overlapped spectra in Figure S23. Using this information, the summary of the proposed mechanism is provided in Figure 6. TPA-ace-TRZ and BPO are both reduced at the electrode surface to produce TPA-ace-TRZ^•–^ and BPO^•–^, respectively. BPO^•–^ then loses a PhCO_2_^–^ group to produce the benzoate radical (PhCO_2_^•^). The benzoate radical then removes an electron from the orbital of TPA-ace-TRZ^•–^ to generate the excited state TPA-ace-TRZ*, which subsequently forms an exciplex species with another PhCO_2_^•^ molecule. The relaxation of this dimeric excited state back to ground state results in the emission of ECL.

The ECL properties of TPA-ace, TPA-ace-CN, and 2TPA-ace were similarly studied (Table 1). In general, the ECL emission wavelengths between annihilation and coreactant pathways were very comparable. The ECL emission wavelengths were the shortest for TPA-ace-CN, suggesting the formation of a higher-energy excited state. The short-wavelength ECL emission may also be responsible for the highest ECL efficiency observed for this compound (relative to a [Ru(bpy)_3_]^2+^ standard), as the 500 nm emission from TPA-ace-CN falls into the most sensitive wavelength range of our photodetector.

### Determination of the Absolute ECL Quantum Efficiency

The absolute ECL quantum efficiency (Φ_ECL_) of TPA-ace-TRZ was then determined to be 0.028% (Table 2), using our standardized spectrograph/CCD camera setup.^63^ The Φ_ECL_ of TPA-ace-TRZ is approximately 9-fold stronger compared to the reference system of [Ru(bpy)_3_]^2+^/BPO. It is evident that there is a large discrepancy between the absolute and relative ECL efficiencies of this system. This is likely because the radical ion stabilities and reactivities of TPA-ace-TRZ and [Ru(bpy)_3_]^2+^ are quite different, and a direct comparison between these systems becomes problematic. In addition, there may be significant quenching of the excited states in the [Ru(bpy)_3_]^2+^/BPO system (particularly at the 10 mM concentrations we have employed in this study), leading to an artificial enhancement of the TPA-ace-TRZ relative efficiencies, wherein a reduction in the denominator of eq S3 increases the relative ECL yield. A key advantage of the absolute ECL determination is significant simplification of the physical/analytical procedure. Only a single measurement for the ECL emission on the spectrograph/CCD apparatus together with the corresponding electrochemical current is needed. In contrast, the relative ECL efficiency always requires a separate measurement of the Ru(bpy)_3_^2+^ standard. This increases the complexity of the test and introduces another potential area of experimental bias. Traditional efficiencies measured relative to an external standard are consequently prone to misrepresentation of the true ECL efficiency. Therefore, reporting the absolute ECL efficiencies provides a highly representative measure of the ECL performance of a luminophore, and allows the meaningful comparison of the performance of various classes of luminophores. Although absolute ECL efficiencies have not been reported before for this class of compound, such measurements can provide valuable insight into their electrochemical and electrochemiluminescence properties and behavior.

**Table 2:** Absolute and Relative ECL Quantum Efficiencies for TPA-ace-TRZ in the Presence of 10 mM Benzoyl Peroxide as Coreactant^a^

	photons	electrons	Φ_ECL_ (%)	rel. efficiency vs [Ru(bpy)_3_]^2+^
[Ru(bpy)_3_]^2+^	4.15 × 10^11^	1.31 × 10^16^	0.003	
TPA-ace-TRZ	4.73 × 10^12^	1.68 × 10^16^	0.028	9-fold

a The concentration was 1.5 mM for both luminophores and calculated during a cyclic voltammogram experiment (scan rate was 0.1 V/s).

### Chemiluminescence and Its Absolute Quantum Efficiency

**Figure 7:**
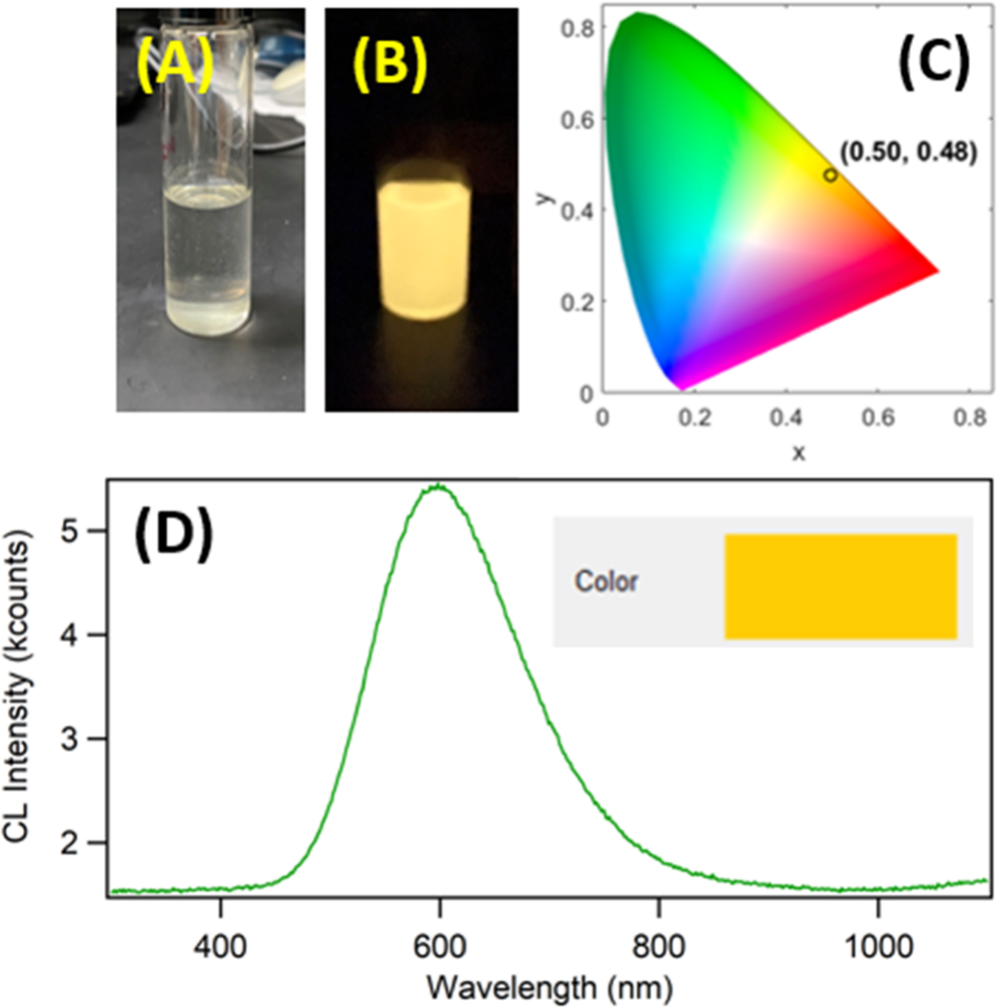
Color photograph of TPA-ace-TRZ CL reaction vial (A) before adding H_2_O_2_ and (B) after addition of H_2_O_2_. (C) CIE color coordinate diagram of the TPA-ace-TRZ CL reaction (0.3 mg/mL TPA-ace-TRZ, 10 mg/mL CPPO, 3 mL 30% H_2_O_2_). (D) Accumulation CL spectrum of 0.3 mg/mL TPA-ace-TRZ in dichloromethane; accumulation time was 10 s. The inset shows the calculated color of the CL emission represented by coordinates *x* = 0.50 and *y* = 0.48.

Finally, we explored the chemiluminescence (CL) properties of TPA-ace-TRZ via the *oxidation of a phenyl oxalate ester*. CL differs from ECL as the energy required to produce emissive excited state species comes from chemical reactions instead of an electrical current. With the addition of 10 mg/mL bis(2,4,5-trichloro-6-(pentyloxycarbonyl)phenyl)oxalate (CPPO) and hydrogen peroxide, bright yellow CL emission can be observed from the reaction vial (Figure 7A,B). Briefly, CPPO reacts with H_2_O_2_ in the presence of a base catalyst (in these experiments, sodium salicylate was used) to produce oxalyl chloride, forming the high-energy 1,2-dioxetanedione intermediate, which ultimately decomposes into CO_2_. This reaction is exothermic, and the released energy can be subsequently absorbed by TPA-ace-TRZ to produce TPA-ace-TRZ*;^24,25,64^ the relaxation of TPA-ace-TRZ* back to ground state releases a CL photon.

The CL emission, when shown on the Commission International de l’Éclairage (CIE) color diagrams, has coordinates of *x* = 0.50 and *y* = 0.48 (Figure 7C); this calculated color matches very well with our photograph. The CL light was also measured by the spectrograph/CCD camera setup, where a broad emission peak could be observed centered at 610 nm (Figure 7D). This signal closely matches the emission observed in the ECL annihilation and coreactant pathways, suggesting that similar excited states—specifically higher-order conjugated excited states—could be present here. However, the presence of excimers and/or exciplexes in the chemiluminescence process is highly intriguing, as the involvement of a secondary coreactant or molecule is not immediately obvious from the reaction mechanism.^25^ We propose that the high-energy intermediate formed between the reaction of CPPO and H_2_O_2_ (pentyl 3,5,6-trichlorosalicylate) may be sufficiently long-lived and have sufficient reactivity to form an exciplex with TPA-ace-TRZ*. In this way, the formation of a dimeric excited state is achieved, analogous to the excited states previously observed in the ECL annihilation and coreactant pathways. Dimeric excited states in chemiluminescence are quite rare, with only a few select examples reported in the literature;^65−67^ this is in stark contrast to the increasingly widespread excimer and exciplex contribution in ECL pathways. It may be that the electro-generation of small, localized pockets of radical species in the vicinity of an electrode are ideal conditions for the formation of excimers and exciplexes. In contrast, CL involves the bulk mixing of reagents, and radical stability and reactivity may be limiting factors. For dimeric excited species to produce the dominant emission, as observed in our CL system, there should be a good balance—both chemically and stoichiometrically—between the luminophore and other reagents to generate the required radical species in sufficient quantities.

Nonetheless, the same excited state of TPA-ace-TRZ is shown to be easily accessible using very different modes of excitation: ultraviolet light in PL (Figure S24), electro-generation of radical species followed by electron transfer in ECL, and decomposition of high-energy chemical intermediates in CL. The overall flexibility and efficiency of TPA-ace-TRZ under these various emissive pathways suggest that it could be a promising luminophore in many light-emitting applications.

**Figure 8:**
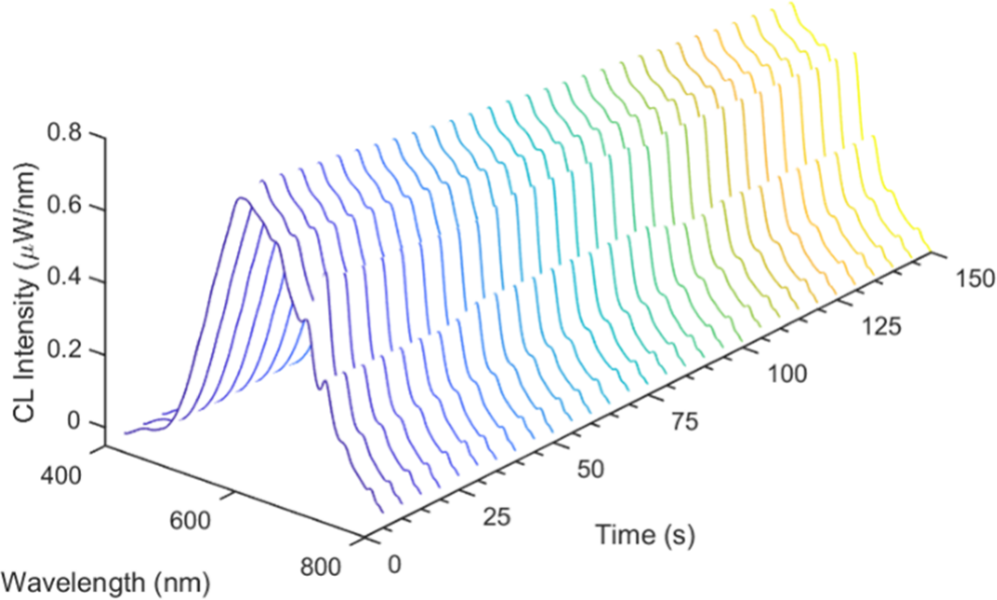
Spooling CL spectra of TPA-ace-TRZ with CPPO and H_2_O_2_ collected using a calibrated Ocean Insight optical spectrometer. Each spectrum is a 5 s exposure.

This reaction was also monitored using spooling CL spectroscopy, a technique that examines the chemiluminescence emission of the system over time. Figure 8 shows the spooling CL spectra, where each spectrum was collected for 10 s for a total of 150 s. Such an acquisition, measured using an externally calibrated spectrometer, enables the quantitative determination of the chemiluminescence quantum yield (Φ_CL_), that is, the number of photons produced per molecule of CPPO (the limiting species in the CL reaction). After integration of each spectrum (with units of μW/nm) across the wavelength range of interest (400–950 nm), and a transformation using the photon energy (eq 1), the number of photons per spectrum can be determined. Summing each spooling CL spectrum over the entire experiment therefore yields the total number of photons collected.

The Φ_CL_ of TPA-ace-TRZ was determined to be 0.11% over the duration of the 150 s experiment (Figure S25); the total efficiency was estimated to be 0.92% when assuming a linear extrapolation to the CL baseline (Figure S26; further details are in the Supporting Information). The compound TPA-ace-TRZ demonstrated remarkable stability during the CL test, with the emission wavelength remaining consistent throughout the 150 s experiment. This is further proof of the presence of a single excited state, which is the exciplex excited state. TPA-ace-TRZ also exhibited a long lifetime in the CL pathway, providing visible emission to the naked eye during the entire experiment. The CL quantum efficiency of TPA-ace-TRZ is also very comparable to other luminophores such as luminol,^68^ showcasing that this compound may have strong applications for clinical immunoassays,^69,70^ or analyte sensing.^71,72^

## Conclusions

Here, we have studied the electrochemistry, electrochemiluminescence, and chemiluminescence of a through-space charge transfer (TSCT) thermally activated delayed fluorescence (TADF) emitter. In particular, TPA-ace-TRZ was shown to possess excimer excited states in ECL annihilation pathways, and exciplex excited states in ECL coreactant and chemiluminescence pathways; these dimeric excited states caused significant red-shifted emissions compared to photoluminescence and were the reason for the organic long-persistent ECL (OLECL) observed in these emitters. TPA-ace-TRZ exhibited an absolute ECL quantum efficiency and an absolute CL quantum efficiency of 0.028 and 0.92%, respectively, determined using our standardized CCD camera and spectrometer.

## Supporting Information Available

Cyclic voltammograms/ECL–voltage curves, differential pulse voltammograms, and ECL accumulation and spooling spectra for TPA-ace, TPA-ace-CN, and 2TPA-ace (PDF)
