# Supplementary material for: Elucidation of an Aggregate Excited State in the Electrochemiluminescence and Chemiluminescence of a Thermally Activated Delayed Fluorescence (TADF) Emitter
Source: Langmuir. 2023 Feb 10;39(7):2829–37. doi: 10.1021/acs.langmuir.2c03391 (PMC9948541; doi:10.1021/acs.langmuir.2c03391)
Supplement: Supplementary file 1 — la2c03391_si_001.pdf [file la2c03391_si_001.pdf]

Supporting information to

# Elucidation of an aggregate excited state in the electrochemiluminescence and chemiluminescence of a thermally activated delayed fluorescence (TADF) emitter

Kenneth Chu[a||], Jonathan R. Adsetts[a||], Zackry Whitworth[a], Shiv Kumar[b], Eli Zysman-Colman[b*], Zhifeng Ding[a,*]

[a] Department of Chemistry, Western University, London, ON, N6A 5B7, Canada

[b] Organic Semiconductor Centre, EaStCHEM School of Chemistry, University of St. Andrews, St. Andrews, Fife KY16 9ST, UK

* Corresponding authors (Emails: eli.zysman-colman@st-andrews.ac.uk; zfding@uwo.ca)

|| Equally contributed authors

**List of Figures**

**Figure S1.** Accumulation spectrum of TPA-ace-TRZ ECL in the annihilation pathway. ....................S-2

**Figure S2.** CV and ECL voltage curve of TPA-ace in dichloromethane with 0.1 M $TBAPF_6$ as the supporting electrolyte. ....................S-3

**Figure S3.** Differential pulse voltammogram of TPA-ace. ....................S-3

**Figure S4.** ECL-time curves during potential stepping experiments (10 Hz pulse frequency) of TPA-ace ECL in the annihilation pathway. ....................S-3

**Figure S5**. Accumulation ECL spectrum of TPA-ace in the annihilation pathway. ....................S-4

**Figure S6.** CV and ECL voltage curve of TPA-ace-CN in dichloromethane with 0.1 M $TBAPF_6$ as the supporting electrolyte. ....................S-4

**Figure S7.** Differential pulse voltammogram of TPA-ace-CN. ....................S-4

**Figure S8.** ECL-time curves for TPA-ace-CN ECL in the annihilation pathway. ....................S-5

**Figure S9.** ECL accumulation spectrum for TPA-ace-CN in the annihilation pathway. ....................S-5

**Figure S10.** CV and corresponding ECL voltage curve of 2TPA-ace with 0.1 M $TBAPF_6$ as supporting electrolyte. ....................S-5

**Figure S11.** Differential pulse voltammogram of 2TPA-ace. ............................................................S-5
**Figure S12.** ECL-time curves during potential stepping experiment (10 Hz) for 2TPA-ace in the annihilation pathway. ..........................................................................................................S-6
**Figure S13.** Accumulation ECL spectrum for 2TPA-ace in the annihilation pathway during potential pulsing experiments....................................................................................................................S-6
**Figure S14.** CV and ECL voltage curve of TPA-ace with 10 mM BPO as coreactant. .......................S-6
**Figure S15.** Accumulation ECL spectrum of TPA-ace with 10 mM BPO. ........................................S-7
**Figure S16.** Spooling ECL spectrum of TPA-ace with 10 mM BPO as coreactant. Scan rate = 0.1 V/s, 1 spectrum/s......................................................................................................................S-7
**Figure S17.** CV and ECL voltage curve for TPA-ace-CN with 5 mM BPO as coreactant...................S-7
**Figure S18.** ECL accumulation spectrum for TPA-ace-CN with 5 mM BPO as coreactant.................S-8
**Figure S19**. ECL spooling spectrum of TPA-ace-CN with 5 mM BPO as coreactant. Scan rate was 0.1 V/s, 1 spectrum/s. ........................................................................................................S-8
**Figure S20.** CV and ECL voltage curve for 2TPA-ace in the presence of 5 mM BPO as coreactant. .S-8
**Figure S21**. ECL accumulation spectrum of 2TPA-ace with 5 mM BPO...........................................S-9
**Figure S22.** Spooling ECL spectra of 2TPA-ace with 5 mM BPO. Scan rate = 0.1 V/s, 1spectrum/s.S-9
**Figure S23.** Overlaid ECL spooling spectra of TPA-ace-TRZ with 10 mM BPO as coreactant........S-10
**Figure S24.** Photoluminescence excitation and emission spectra for TPA-ace-TRZ in dichloromethane. .......................................................................................................................................S-10
**Figure S25.** Total photons per spectrum calculated from the spooling CL experiment of TPA-ace-TRZ with CPPO and $H_2O_2$......................................................................................................S-10
**Figure S26.** Linear extrapolation of the total CL photons to baseline. Linear fit performed using curve fitting function....................................................................................................................S-11

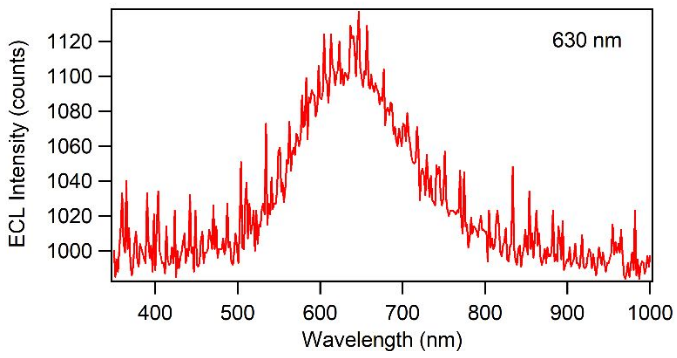


**Figure S1.** Accumulation spectrum of TPA-ace-TRZ ECL in the annihilation pathway.

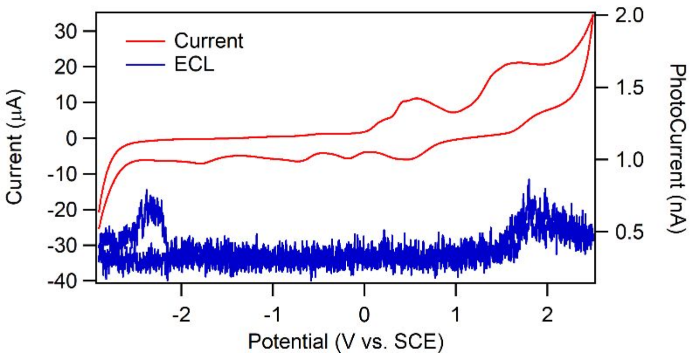


**Figure S2.** CV and ECL voltage curve of TPA-ace in dichloromethane with 0.1 M $TBAPF_6$ as the supporting electrolyte.

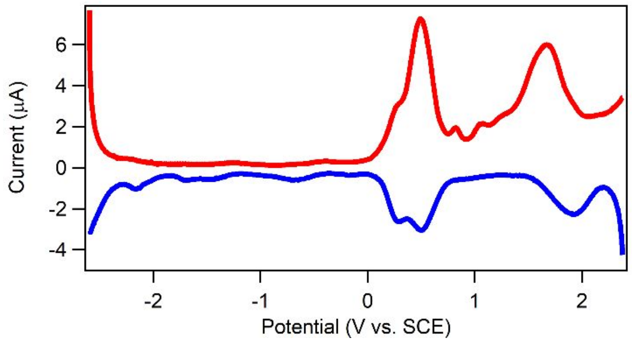


**Figure S3.** Differential pulse voltammograms of TPA-ace.

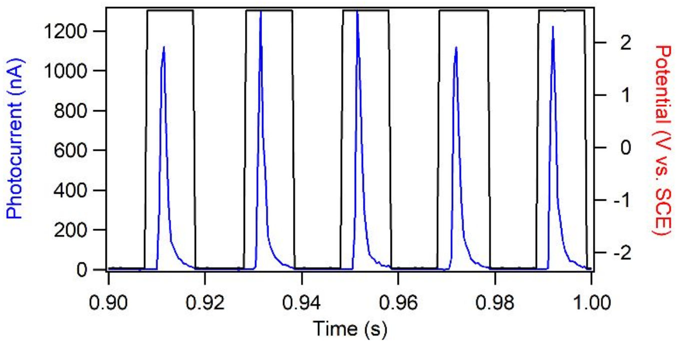


**Figure S4.** ECL-time curves during potential stepping experiments (10 Hz pulse frequency) of TPA-ace ECL in the annihilation pathway.

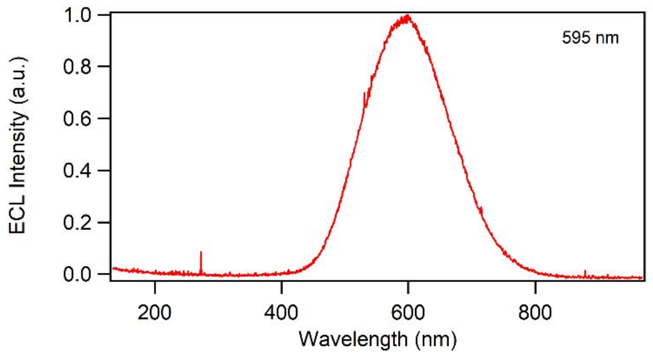


**Figure S5**. Accumulation ECL spectrum of TPA-ace in the annihilation pathway.

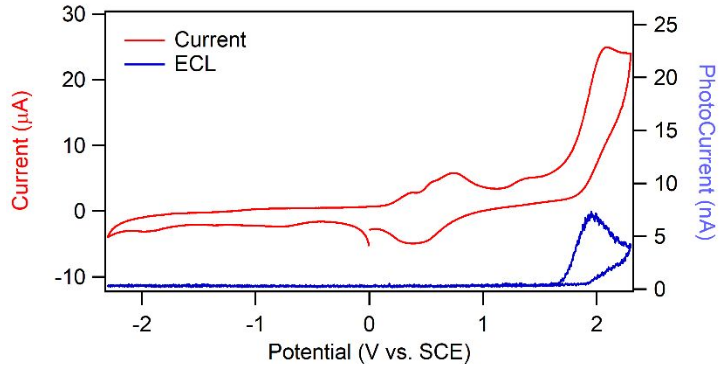


**Figure S6.** CV and ECL voltage curve of TPA-ace-CN in dichloromethane with 0.1 M $TBAPF_6$ as the supporting electrolyte.

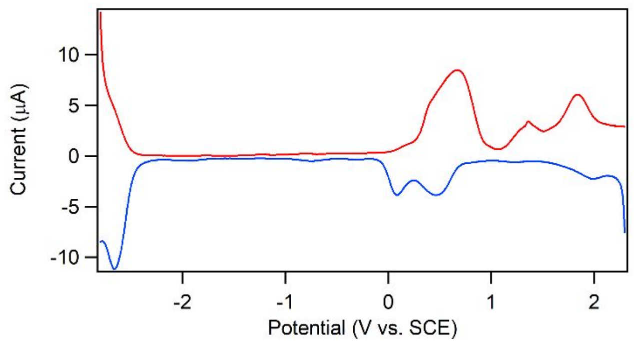


**Figure S7.** Differential pulse voltammograms of TPA-ace-CN.

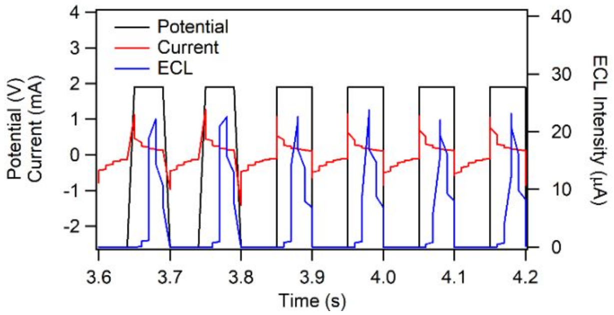


**Figure S8.** ECL-time curves for TPA-ace-CN ECL in the annihilation pathway.

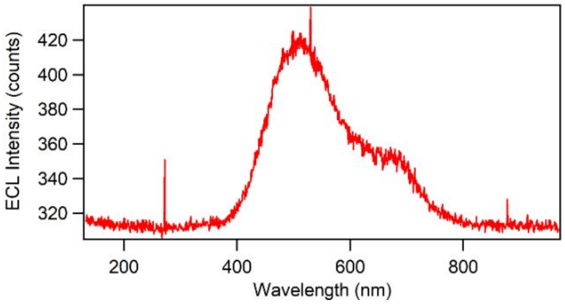


**Figure S9.** ECL accumulation spectrum for TPA-ace-CN in the annihilation pathway.

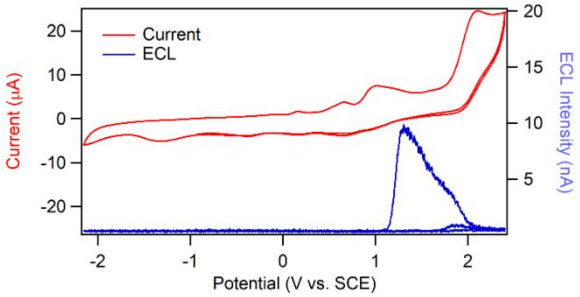


**Figure S10.** CV and corresponding ECL voltage curve of 2TPA-ace with 0.1 M $TBAPF_6$ as supporting electrolyte.

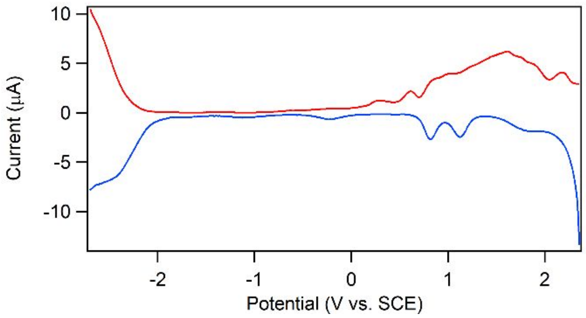


**Figure S11.** Differential pulse voltammograms of 2TPA-ace.

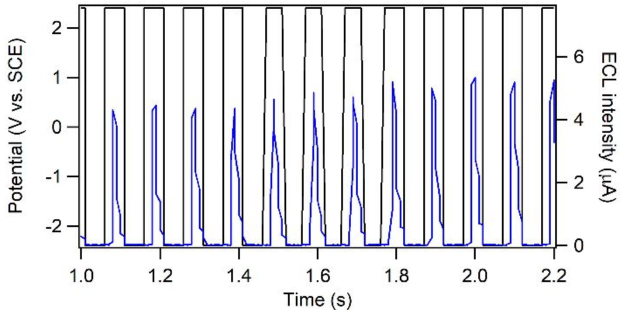


**Figure S12.** ECL-time curves during potential stepping experiment (10 Hz) for 2TPA-ace in the annihilation pathway.

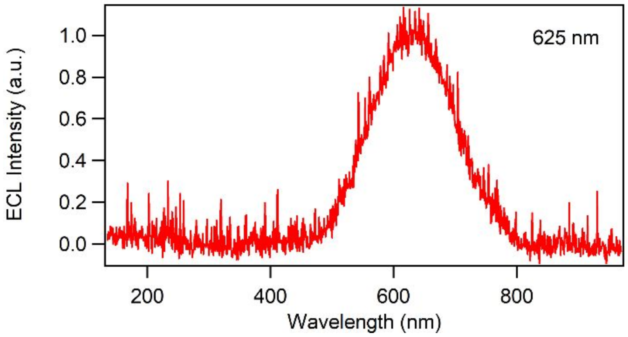


**Figure S13.** Accumulation ECL spectrum for 2TPA-ace in the annihilation pathway during potential pulsing experiments.

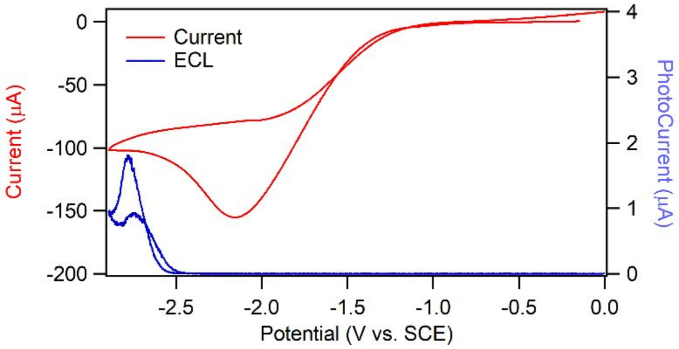


**Figure S14.** CV and ECL voltage curve of TPA-ace with 10 mM BPO as coreactant.

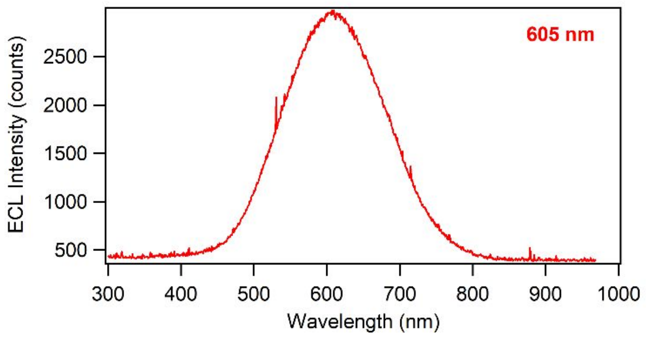


**Figure S15.** Accumulation ECL spectrum of TPA-ace with 10 mM BPO.

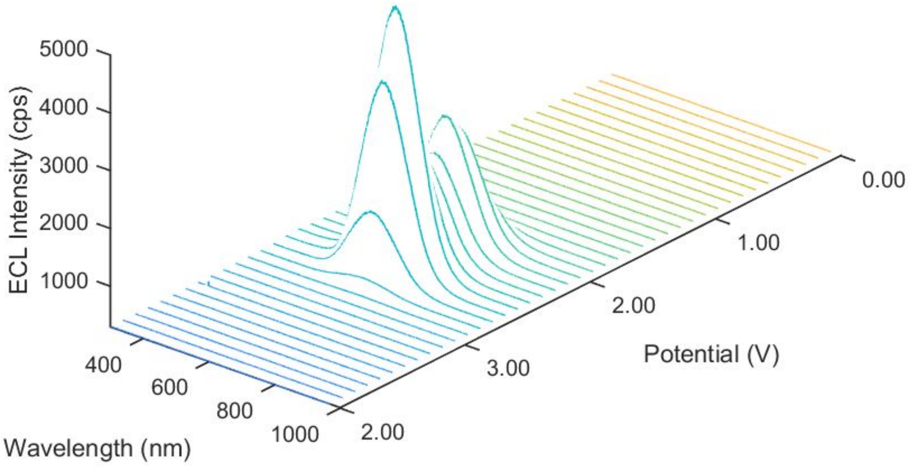


**Figure S16.** Spooling ECL spectrum of TPA-ace with 10 mM BPO as coreactant. Scan rate = 0.1 V/s, 1 spectrum/s.

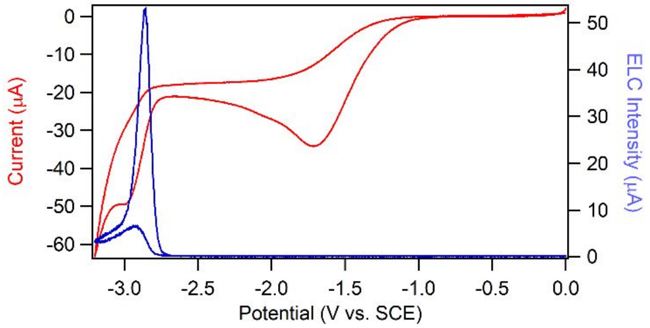


**Figure S17.** CV and ECL voltage curve for TPA-ace-CN with 5 mM BPO as coreactant.

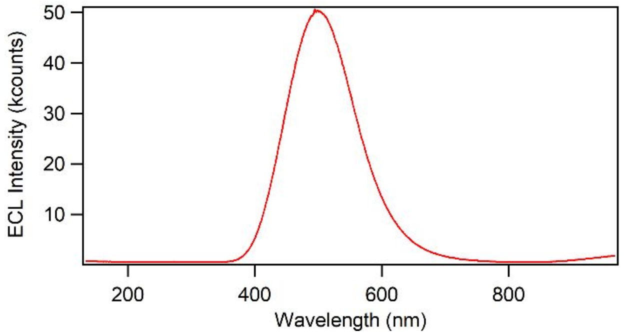


**Figure S18.** ECL accumulation spectrum for TPA-ace-CN with 5 mM BPO as coreactant.

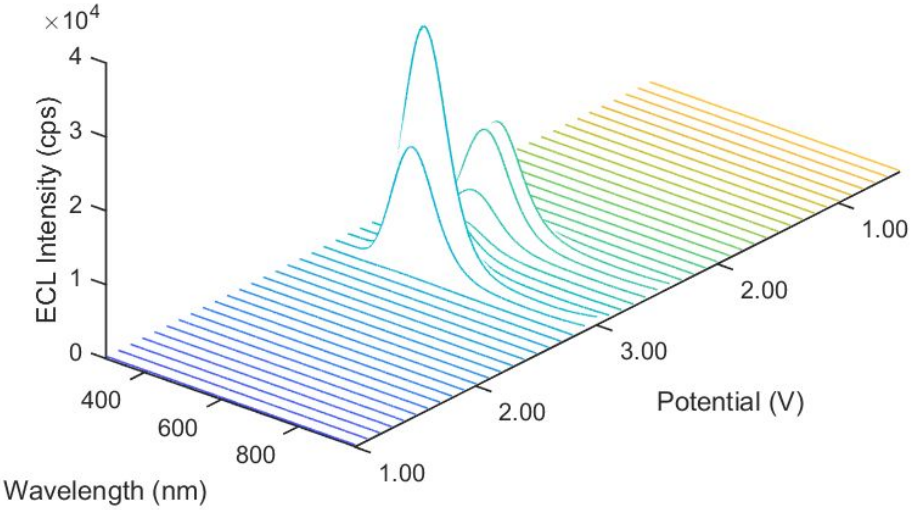


**Figure S19**. ECL spooling spectrum of TPA-ace-CN with 5 mM BPO as coreactant. Scan rate was 0.1 V/s, 1 spectrum/s.

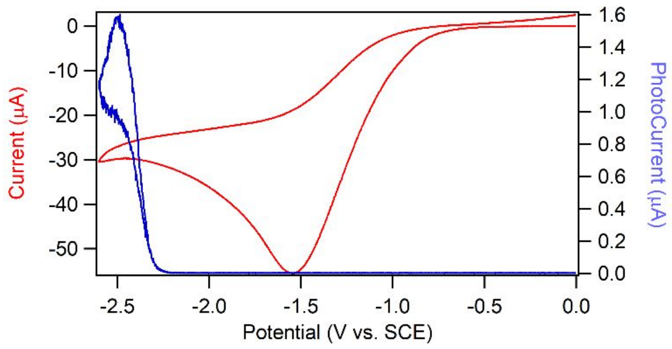


**Figure S20.** CV and ECL voltage curve for 2TPA-ace in the presence of 5 mM BPO as coreactant.

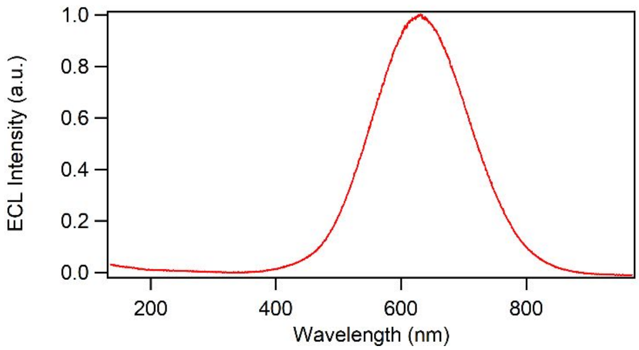


**Figure S21**. ECL accumulation spectrum of 2TPA-ace with 5 mM BPO.

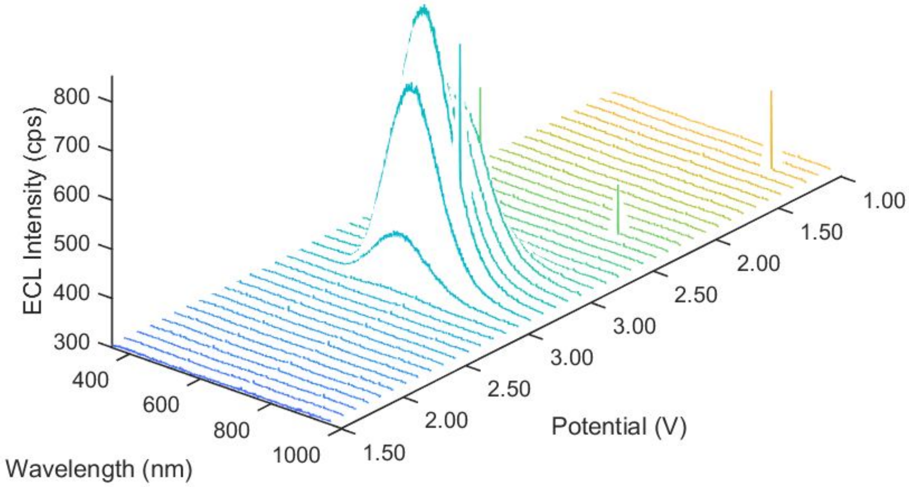


**Figure S22.** Spooling ECL spectra of 2TPA-ace with 5 mM BPO. Scan rate = 0.1 V/s, 1 spectrum/s.

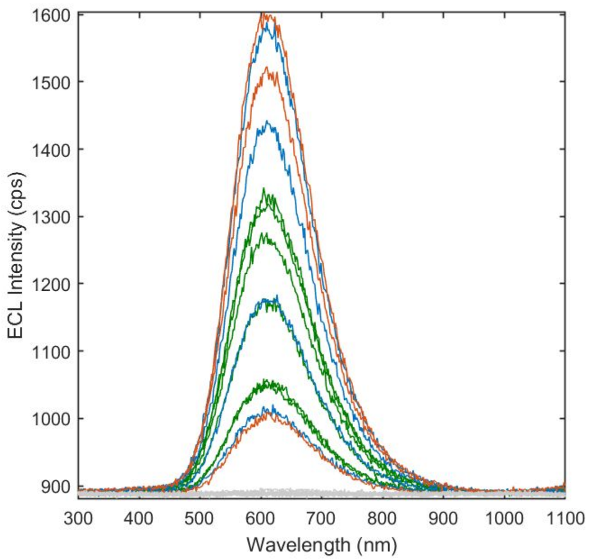


**Figure S23.** Overlaid ECL spooling spectra of TPA-ace-TRZ with 10 mM BPO as coreactant.

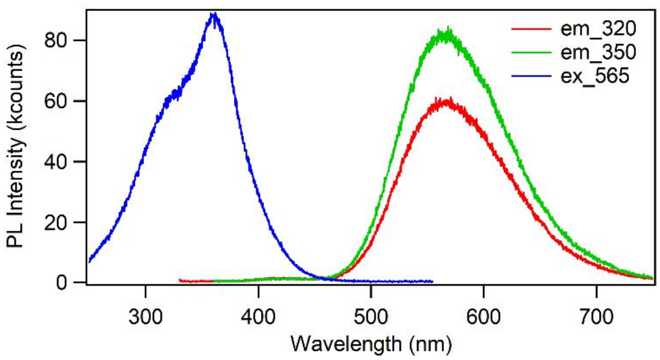


**Figure S24.** Photoluminescence excitation and emission spectra for TPA-ace-TRZ in dichloromethane.

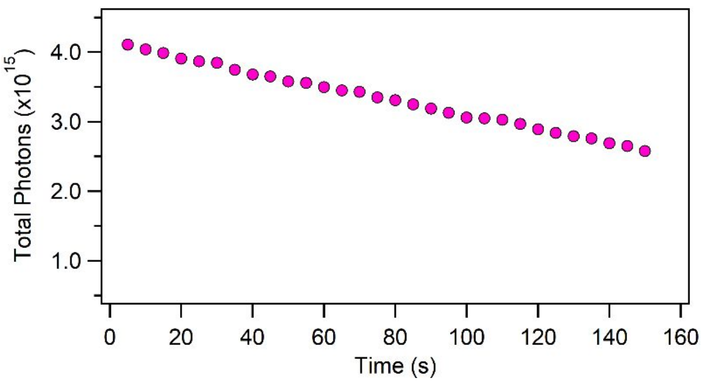


**Figure S25.** Total photons per spectrum calculated from the spooling CL experiment of TPA-ace-TRZ with CPPO and $H_2O_2$.

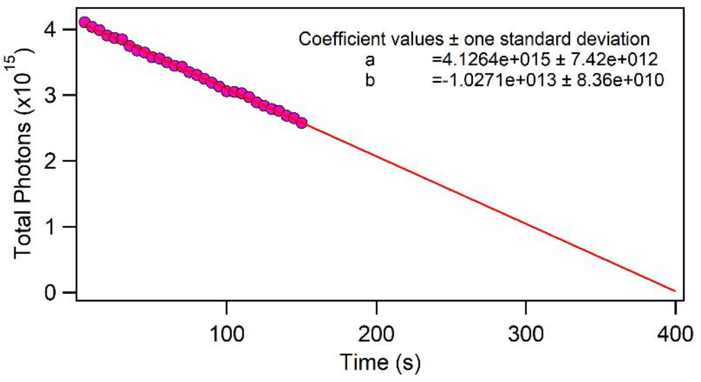


**Figure S26.** Linear extrapolation of the total CL photons to baseline. Linear fit performed using curve fitting function.

**Estimation of the total number of photons in TPA-ace-TRZ CL experiment.** An estimation of the total CL photons curve was performed by extrapolating the linear fit to the x-intercept (when the total photons curve becomes zero). At this point, t = 400 s. The number of photons is therefore the area under the above plot. Calculating the area:

$$Area = \frac{base \times height}{2} = \frac{400\ s \times 4.11 \times 10^{15}}{2} = 8.22 \times 10^{17}\ photons\ \#(1)$$

$$\Phi_{CL} = \frac{total\ photons}{n_{CPPO} \times NA} \times 100 = \frac{8.22 \times 10^{17}}{1.48 \times 10^{-4} \times 6.02 \times 10^{23}} \times 100 = 0.92\%\ \#(2)$$

**Equation S3.** Calculation for the relative ECL efficiency, where *x* is the luminophore under study and *st* is the standard $[Ru(bpy)_3{}^{2+}]$.

$$\text{rel. } \Phi_{ECL} = \frac{\left(\frac{\int ECL \times dt}{Current \times dt}\right)_x}{\left(\frac{\int ECL \times dt}{\int Current \times dt}\right)_{st}} \times 100\ \#(3)$$

**Table S1.** Summary of ECL onset, peak maxima, and end during ECL pulsing experiments.

| | **1. TPA-ace** | **2. 2TPA-ace** | **3. TPA-ace-CN** | **4. TPA-ace-TRZ** |
|---|---|---|---|---|
| **$\Delta E_{ST}$[1]** | 0.88 eV | 0.74 eV | 0.22 eV | 0.06 eV |
| **ECL start delay** | +23 ± 2.5 ms | +20 ± 1.8 ms | +18 ± 2.4 ms | +25 ± 3.7 ms |
| **ECL max** | +34 ± 3.5 ms | +31 ± 2.4 ms | +32 ± 3.9 ms | +57 ± 10 ms |
| **ECL end** | +111 ± 14 ms | +100 ± 1.8 ms | +100 ± 1.0 ms | +128 ± 14 ms |

ECL Time offsets were determined from ECL-time profiles during potential pulsing experiments (as in Figure 3C, S4, S8, and S12 corresponding to TPA-ace-TRZ, TPA-ace, TPA-ace-CN, and 2TPA-ace, respectively. 'ECL start delay' corresponds to the beginning of the increase from the ECL baseline. 'ECL max' corresponds to the point of maximum recorded ECL intensity. 'ECL end' corresponds to the point where the ECL returns to the baseline. All time offsets are reported with respect to the beginning of the potential pulse (t=0).

## References

[1] Kumar, S.; Franca, L. G.; Stavrou, K.; Crovini, E.; Cordes, D. B.; Slawin, A. M. Z.; Monkman, A. P.; Zysman-Colman, E. Investigation of Intramolecular Through-Space Charge-Transfer States in Donor–Acceptor Charge-Transfer Systems. *J. Phys. Chem. Lett.* **2021,** *12*, 2820-2830.
